# Soluble CD26/Dipeptidyl Peptidase IV Enhances the Transcription of IL-6 and TNF-α in THP-1 Cells and Monocytes

**DOI:** 10.1371/journal.pone.0066520

**Published:** 2013-06-21

**Authors:** Tetsurou Ikeda, Emi Kumagai, Satoshi Iwata, Akio Yamakawa

**Affiliations:** University of Tokyo, Institute of Medical Science, Tokyo, Japan; University of Leuven, Rega Institute, Belgium

## Abstract

CD26 is a 110-kDa multifunctional molecule having dipeptidyl peptidase IV (DPPIV) enzyme activity and is present on the surface of human T cells. Soluble CD26 (sCD26) exists in human blood and enhances the proliferation of peripheral T lymphocytes induced by tetanus toxoid (TT). The mechanisms by which CD26 enhances the activation of T cells and monocytes remain to be fully elucidated. In this study, we compared the stimulation of THP-1 cells and isolated human monocytes with a combination of recombinant sCD26 and lipopolysaccharide (LPS) and the stimulation of these cells with LPS alone. We found that addition of sCD26 increased TNF-α and IL-6 mRNA and protein expression and enhanced ERK1/2 levels in the cytosol as well as c-Fos, NF-κB p50, NF-κB p65, and CUX1 levels in the nuclei of these cells. On the other hand, the selective DPPIV inhibitor sitagliptin inhibited the increase in TNF-α mRNA and protein expression as well as the increase in ERK, c-Fos, NF-κB p50, NF-κB p65, and CUX1 levels. However, it did not inhibit the increase in IL-6 mRNA and protein expression. We then demonstrated that sCD26 enhanced binding of transcription factors to the TNF- and IL-6 promoters and used reporter assays to demonstrate that transcription factor binding enhanced promoter activity. Once again, we observed differential activities at the TNF- and IL-6 promoters. Finally, we demonstrated that CUX-1 overexpression enhanced TNF- production on sCD26/LPS stimulation, while CUX-1 depletion had no effect. Neither CUX-1 overexpression nor CUX-1 depletion had an effect on IL-6 stimulation. These results are discussed in the context of a model that describes the mechanisms by which stimulation of monocytic cells by sCD26 and LPS leads to elevation of TNF- and IL-6 expression. CUX-1 is identified as a new transcription factor that differently regulates the activities of the TNF- and IL-6 promoters.

## Introduction

CD26 is a 110-kDa cell surface glycoprotein with known dipeptidyl peptidase IV (DPPIV, EC 3.4.14.5) activity in its extracellular domain [1], [2], [3] capable of cleaving N-terminal dipeptides with l-proline or l-alanine at the penultimate position [3]. CD26 has two physiological forms in humans [3]. One of them (CD26) is a membrane-bound form preferentially expressed on the CD4^+^ helper/memory T cell population, while the other (sCD26) is a soluble form found in serum. Cross-linking of CD26 and CD3 with solid-phase immobilized monoclonal antibodies (mAbs) can induce T cell costimulation and interleukin (IL)-2 production by human CD4^+^ T cells or Jurkat T cell lines transfected with CD26 cDNA [3], [4], [5]. Furthermore, anti-CD26 antibody treatment of T cells results in a decrease in the surface expression of CD26 through its internalization. This modulation results in an enhanced proliferative response to anti-CD3 or anti-CD2 stimulation as well as an enhanced tyrosine phosphorylation of signaling molecules such as CD3 and p56lck [5]. Moreover, we have shown that DPPIV enzyme activity is required for CD26-mediated T cell costimulation [6]. In a recent study, we demonstrated that caveolin-1 binds to CD26 and that CD26 on activated memory T cells interacts with caveolin-1 on tetanus toxoid (TT)-loaded monocytes [7]. We also identified caveolin-1 on antigen-presenting cells (APC) and demonstrated that CD26 stimulation upregulates surface expression of CD86 on APC through caveolin-1 and enhances TT-mediated T-cell proliferation [7]. The signaling pathways stimulated by CD26-mediated phosphorylation of caveolin-1 (p-cav-1), which eventually lead to the upregulation of CD86 in APC, are yet to be elucidated. More recently, we demonstrated that caveolin-1 binds to Toll-interacting protein (Tollip) and IL-1 receptor associated serine/threonine kinase 1 (IRAK-1) in the membrane of TT-loaded monocytes. Following exogenous CD26 stimulation, Tollip and IRAK-1 disengage from caveolin-1, with IRAK-1 being subsequently phosphorylated to upregulate CD86 expression [8]. It is conceivable that the interaction of CD26 with caveolin-1 on antigen-loaded monocytes results in CD86 upregulation, thereby enhancing the subsequent interaction of CD86 and CD28 on T cells to induce antigen-specific T-cell proliferation and activation. However, the precise mechanism by which sCD26 enhances LPS (liposaccharide)-Toll-like receptor 4 signaling in monocytic cells is still unresolved. In this study, we demonstrated that stimulation of THP-1 cells and purified human monocytes with a combination of sCD26 and LPS enhanced the expression of TNF-α and IL-6 mRNA and protein compared with stimulation by LPS alone. Importantly, we also found that stimulation with a combination of sCD26 and LPS enhanced the expression of c-Fos, NF-κB p65, NF-κB p50, and CUX1 in THP-1 cells and monocytes. These results suggest that sCD26 can be useful in potentiating an innate immune response in selected clinical settings.

## Materials and Methods

### Cells and Antibodies

This study was conducted according to the Declaration of Helsinki. Experimental protocols were approved by the Ethics Committee of the University of Tokyo, from where all participants were recruited, under informed written consent, and human experimentation was conducted.

THP-1 cells were grown as described previously in *Molecular and Cellular Biology* [8]. Human monocytes were purified from peripheral blood mononuclear cells (PBMCs) collected from healthy adult volunteers after documented informed consent was obtained. A MACS human monocytes isolation kit (Miltenyi Biotec) was used to purify the human monocytes. Nonmonocytes, namely CD4+ T cells, CD8+ T cells, neutrophils, eosinophils, B cells, stem cells, dendritic cells, NK cells, granulocytes, **γ**/**δ** T cells, or erythroid cells were specifically depleted using antibodies against CD4, CD15, CD16, CD19, CD34, CD36, CD56, CD123, TCR**γ**/**δ**, and CD235a. Purity of the monocytes was ≥90%, as confirmed by FACSCalibur (BD Biosciences). Other antibodies used for flow cytometry were purchased from BD Biosciences.

### Preparation of sCD26

sCD26 with DPPIV activity (sCD26/DPPIV+) was produced according to the method described previously [9]. In brief, the expression vector RcSRα-263–9, which contains a deletion of the coding sequence for amino acids 3–9 of CD26, was transfected into the dihydrofolate reductase-deficient Chinese hamster ovary (CHO) cell line DXB-11 by electroporation, together with plasmid pMT-2, which provided the dihydrofolate reductase gene. Mutant sCD26 without DPPIV activity (sCD26/DPPIV–) was produced using the same method, except that RcSRα-263–9 was further modified to yield RcSRα-263–9 S630A, which contains a point mutation at the active site of the DPPIV enzyme (Ser630 replaced by Ala) created using oligonucleotide-mediated site-directed mutagenesis. The transfected CHO cells, which produce either sCD26 or mutant sCD26, were cultured in serum-free CHO-S-SFM II medium (Life Technologies) supplemented with 1 µM methotrexate (Sigma-Aldrich). The culture supernatant was collected and subjected to affinity chromatography on adenosine deaminase-Sepharose according to the methods described previously [10].

### Characterization of sCD26 Proteins and the DPPIV Inhibitor Sitagliptin

To investigate the role of DPPIV activity in sCD26 function, we expressed two different forms of sCD26 protein in CHO cells and purified them by affinity chromatography. The first construct CD26/DPPIV(+) is a wild-type protein, while the second construct sCD26/DPPIV(−) contains a serine-to-alanine mutation at residue 630, which is a key residue in the DPPIV active site. Both purified proteins migrate as a single band at approximately 110 kDa on an SDS-PAGE gel. However, the DPPIV activity of sCD26/DPPIV(+) is approximately 100-fold greater than that of sCD26/DPPIV(−). We tested the DPPIV inhibitors diprotin A, P32/98, and sitagliptin as inhibitors of the DPPIV activity of sCD26/DPPIV(+) and found them to essentially exhibit the same activity as that reported previously. We used these purified proteins for subsequent stimulation experiments.

### Measurement of Cytokines

Purified monocytes (1×10^5^) and THP-1 cells were incubated with LPS (1 and 10 µg/ml, respectively) and/or sCD26/DPPIV(+) (0.002, 0.02, 0.2, 2, 20 µg/ml) or sCD26/DPPIV(−) (0.002, 0.02, 0.2, 2, 20 µg/ml) in 96-well flat bottom plates for 3, 6, 12, 24, or 36 h. The doses of LPS used in subsequent experiments were determined as the half response levels from each saturation curve (data not shown). After incubation, supernatants were collected and cytokine concentrations were examined using enzyme-linked immunosorbent assays (ELISAs). BD OptEIA kits for human IL-6 and TNF-α were purchased from BD Biosciences.

### Antibodies (Abs) and Reagents

The sources and working concentrations of mAbs used as primary Abs for flow cytometry were as follows: PE-conjugated anti-CD3 (UCHT1, mouse IgG1; 10 µg/ml; BD PharMingen) and anti-CD14 (Mo-2, mouse IgM; 10 µg/ml; Beckman Coulter, Miami, FL). LPS was obtained from Invitrogen. Antibodies used here were as follows: Lamin A/C (sc20681 Santa Cruz), CUX1 (M-222, sc-13024), NF-κB p50 (sc-1192), NF-κB p65 (sc372), c-Fos (sc-52), and PolII (N-20, sc-899), all from Santa Cruz. GAPDH (ab8245-100 Abcam), ERK (610124, BD), and AKT (610877, BD) were also used. DPPIV inhibitors were P32/98 (Biomol), diprotin A (Biomol), and sitagliptin (Merck).

### DNA Construction

Full-length human CUX1/CUTL1 in pMX (CUX1/CUTL1 p200) and pXJ42 as well as the sh-RNA for mouse and human CUX1 (5′-AAGAAGAACACTCCAGAGGATTT-3′), which was subcloned into pSuper.retro.puro, were kind gifts from Dr. Downward. Mismatched sh-CUX at four nucleotides was prepared to examine nonspecific effects (mis-sh-CUX1, 5′-UAGAACAACACACCAGACGATTT-3′). Promoters for TNF-α [TNF-α 2-1 (−1.8 kb), TNF-α 3-1 (−1.6 kb), TNF-α 5-1 (−0.6 kb)], and IL-6 [IL-6 (−1.0 kb)] were generated and sequenced and then subcloned into the luciferase vector (PGV-B, TOYO INK).

### Quantitative RT-PCR Assay

Total RNA was isolated from THP-1 cells and monocytes by acid–phenol extraction [11] and used to synthesize cDNA with the SuperScriptIII RNase H^-^ reverse transcriptase kit (Invitrogen) and random hexamer primer (Invitrogen). Quantification of mRNA was performed using the Roche LightCycler 2.0, TaqMan probe, and LightCycler TaqMAN Master (Roche). The data obtained were analyzed using Roche LightCycler 2.0 Software (Roche) and normalized to GAPDH expression. PCR was performed using the following primers: TNF-α forward primer, 5′-GGAGGGGTCTTCCAGCTGGAGA-3′, reverse primer, 5′-CAATGATCCCAAAGTAGACCTGC-3′, probe, 5′-FAM- ACCGACTCAGCGCTGAGATCAATCGGCCCGACTAT-TAMRA-3′; IL-6 forward primer, 5′-CCAGCCTGCTGACGAAGCTGCAGG-3′, reverse primer, 5′-AAGAGCCCTCAGGCTGGACTGCA-3′, probe, 5′-FAM-AGAACCAGTGGCTGCAGGACATGACAACTCATCTC-TAMRA-3′; and GAPDH forward primer, 5′-TGATGACATCAAGAAGGTGGTGAAG-3′, reverse primer, 5′-TCCTTGGAGGCCATGTAGGCCAT-3′, probe, 5′-FAM-ACTTTGTCAAGCTCATTTCCTGGTATGACAA-TAMRA-3′.

### Western Blotting

Nuclear extracts and cytosolic fractions were prepared as described previously [12]. Cells were washed with phosphate buffered saline and solubilized with Laemmli sample buffer. The samples were centrifuged, and the pellet was resuspended in Laemmli sample buffer with beta-mercaptoethanol for SDS-PAGE, followed by Western blotting. SDS-PAGE was performed on 10% polyacrylamide gels, and separated proteins were transferred onto nitrocellulose membranes at 30 V for 90 min or more. Membranes were blocked by incubation for 1 h with 5% nonfat milk in phosphate buffered saline containing 0.2% Tween 20, and antigens were detected using specific antibodies. Membranes were then incubated with appropriate horseradish peroxidase-conjugated bound secondary antibody, and the signals were then detected using an enhanced chemiluminescence protocol (Amersham Biosciences) as described by the manufacturer.

### Luciferase Assays

The activities of the reporter gene luciferase, used in transient transfection assays, were measured using the Dual Luciferase reporter assay system (Promega) according to the manufacturers’ instructions. Lipofectamine 2000 and DMRIE-C (Life Technologies, Invitrogen) were used for transfection, as described in the manufacturers’ protocol.

### Chromatin Immunoprecipitation (ChIP) Assays

ChIP assays were performed on THP-1 cells and monocytes treated, or not treated, with LPS-sCD26(+)/(−) using a commercially available kit (Upstate) according to the manufacturers’ instructions. In brief, the cells were fixed with formaldehyde for 10 min in growth medium to cross-link the protein/DNA complexes. The cells were then lysed, and the nuclei were isolated after sonication to produce soluble chromatin DNA with an average size of 500 bp. Input samples were taken obtained at this point to act as positive controls. The cell lysates were then pre-cleared using salmon sperm DNA/protein agarose slurry. DNA protein complexes were immunoprecipitated with antibodies to Pol II, CUX1, c-Fos, NF-kB p50, or NF-kB p65. Antibody-bound DNA/protein complexes were recovered by centrifugation, washed using buffers of increasing ionic strength, eluted from the antibodies, and proteinase-treated to remove residual protein. Cross-linking was reversed, and the DNA fragments were recovered and used in PCR amplifications with appropriate primers spanning the TNF-α promoter (probe 2 forward primer, 5′-CCGGAGCTTTCAAAGAAGGAATTCT-3′, reverse primer, 5′-CCCCTCTCTCCATCCTCCATAAA-3′, probe, 5′-FAM-CAGCCCAAAGCTGTTGGTCTGTCCCACCAGCTA -TAMRA-3′; probe 3 forward primer, 5′- ACCAAGAGAGAAAGAAGTAGGCATG-3′, reverse primer, 5′-AGCAGTCTGGCGGCCTCACCTGG-3′, probe, 5′-FAM-TCTGGGAGTGAGAACTTCCCAGTCTATCTAAGGAA-TAMRA-3′; probe 5 forward primer, 5′- TTATGAGTCTCCGGGTCAGAATGA -3′; reverse primer, 5′AGACAGGATGCAGGAAAAAGATAG -3′; probe, 5′-FAM CCGCAGGGACCCAAACACAGGCCTCAGGACTCAACA TAMRA-3′; and IL-6 promoter probe 3 forward primer, 5′-GACATGCCAAAGTGCTGAGTCACT-3′, reverse primer, 5′-AGACTCATGGGAAAATCCCACATT-3′, probe, 5′-FAM CATGCTAAAGGACGTCACATTGCACAATCTTAATA -TAMRA-3′).

### Data Analysis

Data are expressed as the mean ± S.E of at least 5–6 independent experiments, each performed in triplicate. Statistical analysis was performed using Student’s *t*-test. Values of *P*<0.05 were considered significant.

## Results

### sCD26 Enhancement of LPS-stimulated TNF-α and IL-6 mRNA Expression in Monocytic Cells

The Toll-like receptor (TLR) signaling pathway plays a central role in innate immunity and is linked to the activation of adaptive immunity. TLRs are activated by interaction with their cognate ligands, such as LPS, followed by the activation of two distinct adaptor molecules, MyD88 and TRIF. The MyD88 and TRIF signaling pathways activate the transcription factors NF-κB and IRF3, leading to the transcriptional activation of proinflammatory cytokines (TNF-α, IL-6, and IL-12) and interferons [13].

**Figure 1:**
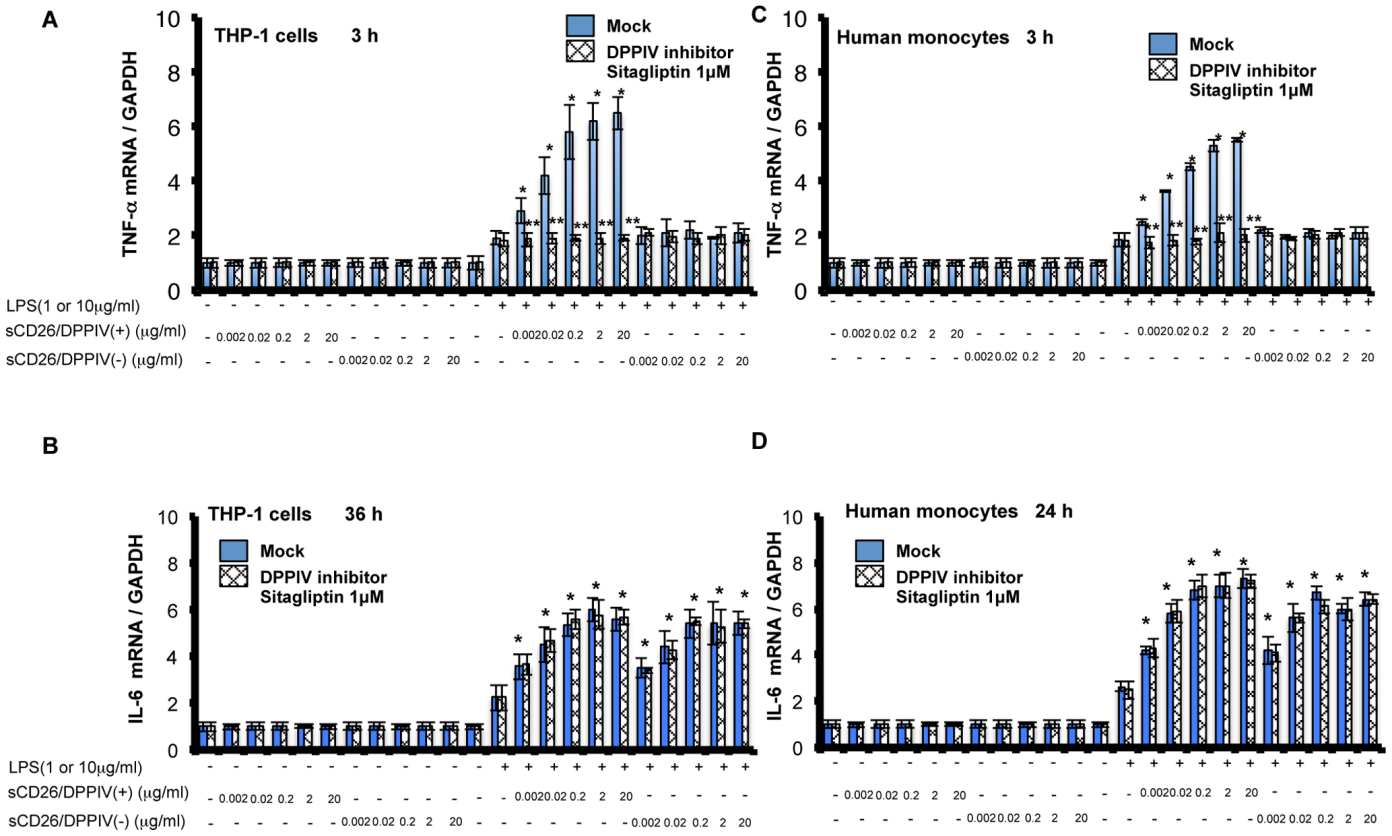
Effects of LPS and sCD26/DPPIV(+) or sCD26/DPPIV(−) stimulation of THP-1 cells and monocytes on TNF-α and IL-6 mRNA expression, assessed by quantitative RT-PCR. **A, B.** Effects of stimulating THP-1 cells with a combination of LPS and sCD26/DPPIV(+) or sCD26/DPPIV(−) on TNF-α and IL-6 mRNA expression. THP-1 cells (1×10^5^) were incubated in the culture medium with or without LPS or various concentrations of sCD26/DPPIV(+) and sCD26/DPPIV(−). The cells were then cultured for the indicated periods. Total RNA was prepared and subjected to quantitative RT-PCR analysis. Each panel shows a typical example of the results. Values represent mean ± S.E.M., n = 3, *P<0.05 vs. control (stimulation with LPS alone). **P<0.05 vs. Mock. C, D. Effects of stimulating monocytes with a combination of LPS and sCD26/DPPIV(+) or sCD26/DPPIV(−) on TNF-α and IL-6 mRNA expression. Monocytes (1×10^5^) were incubated in the culture medium with or without LPS or various concentrations of sCD26/DPPIV(+) or sCD26/DPPIV(−). Then, the cells were cultured for the indicated periods. Total RNA was prepared and subjected to quantitative RT-PCR analysis. Each panel shows a typical example of the results. Values represent mean ± S.E.M., n = 3, *P<0.05 vs. control (stimulation with LPS alone). **P<0.05 vs. Mock. Statistical analysis was performed using Student’s *t-*test.

However, the influence of sCD26 on monocytic cells treated with LPS is unclear. We treated cultured THP-1 cells and isolated human monocytes with both sCD26 and LPS and examined for effects on the levels of TNF-α and IL-6 mRNAs. We found that sCD26/DPPIV(+) enhanced the LPS-stimulated expression of TNF-α mRNA (Figure 1A) and IL-6 mRNA (Figure 1B) in cultured THP-1 cells. In contrast, sCD26/DPPIV(−) did not enhance the LPS-stimulated expression of TNF-α mRNA (Figure 1A) but did enhance the LPS-stimulated expression of IL-6 mRNA (Figure 1B) in THP-1 cells. Consistent with these results, addition of the specific DPPIV inhibitor sitagliptin (1 M) inhibited the sCD26/DPPIV(+)-mediated enhancement of LPS-stimulated TNF-α expression (Figure 1A) but had no effect on the enhancement of LPS-stimulated expression of IL-6 mRNA (Figure 1B). We also found that sCD26/DPPIV(+), but not sCD26/DPPIV(−), enhanced the LPS-stimulated expression of TNF-α mRNA (Figure 1C) in human monocytes. Addition of sitagliptin (1 M) inhibited the sCD26/DPPIV(+)-mediated enhancement of LPS-stimulated TNF-α mRNA expression (Figure 1C). Both sCD26/DPPIV(+) and sCD26/DPPIV(−) enhanced the LPS-stimulated expression of IL-6 mRNA in human monocytes, and addition of sitagliptin (1 M) did not have a significant effect (Figure 1C, D).

### sCD26 Enhancement of LPS-stimulated Secretion of TNF-α and IL-6 Proteins

**Figure 2:**
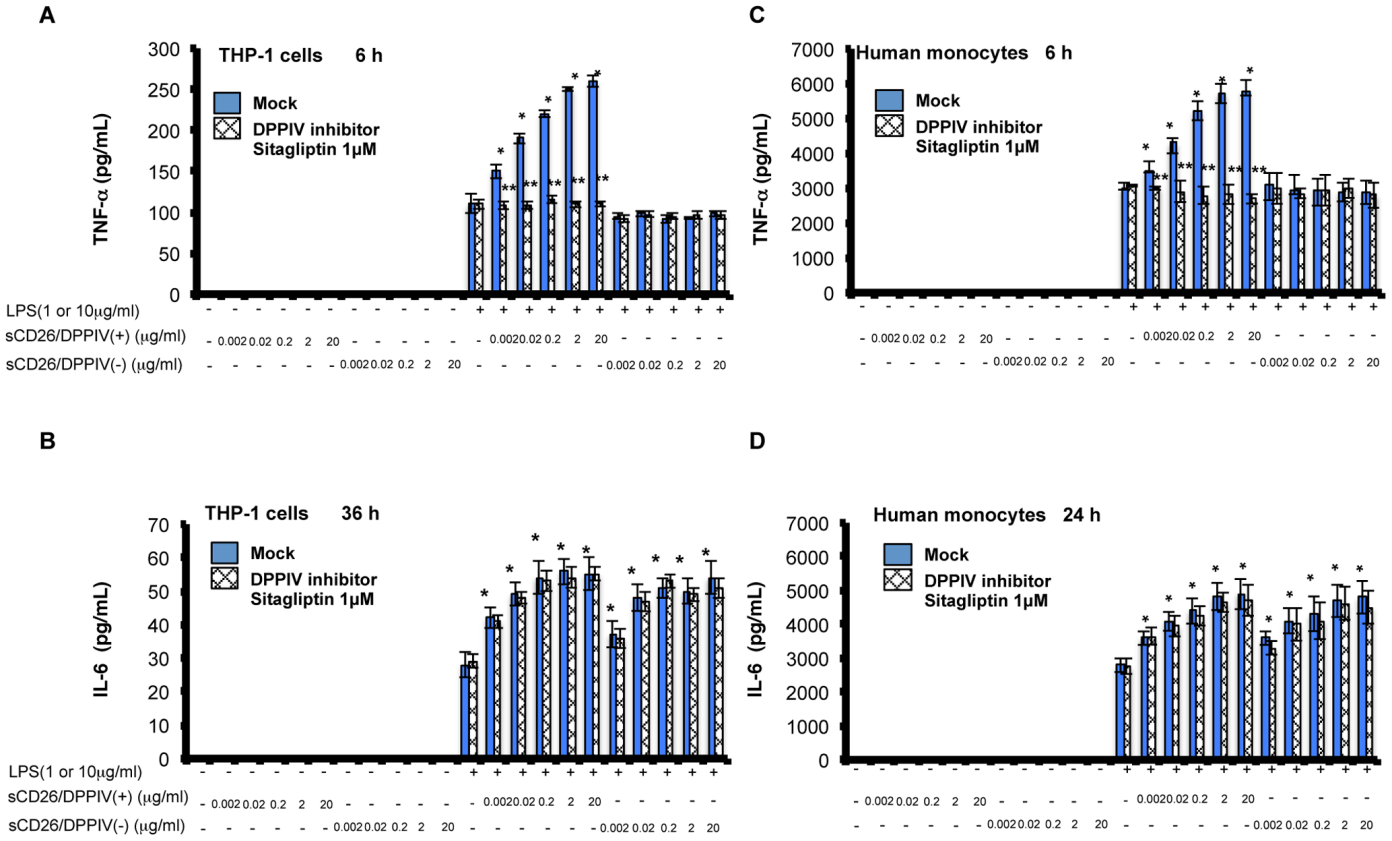
Effects of LPS and sCD26/DPPIV(+) or sCD26/DPPIV(−) stimulation of THP-1 cells and monocytes on TNF-α and IL-6 protein secretion, assessed using ELISAs. A, B. Effects of stimulating THP-1 cells with a combination of LPS and sCD26/DPPIV(+) or sCD26/DPPIV(−) on TNF-α and IL-6 protein secretion. THP-1 cells (1×10^5^) were incubated in the culture medium with or without LPS or various concentrations of sCD26/DPPIV(+) or sCD26/DPPIV(−). The cells were then cultured for the indicated periods. Serum was obtained from the well and subjected to ELISA. Each panel shows a typical example of the results. Values represent mean ± S.E.M., n = 5, *P<0.05 vs. control (stimulation with LPS alone). **P<0.05 vs. Mock. C, D. Effects of stimulating monocytes with a combination of LPS and sCD26/DPPIV(+) or sCD26/DPPIV(−) on TNF-α and IL-6 protein secretion. Monocytes (1×10^5^) were incubated in the culture medium with or without LPS or various concentrations of either sCD26/DPPIV(+) or sCD26/DPPIV(−). The cells were then cultured for the indicated periods. Serum was obtained from the well and subjected to ELISA. Each panel shows a typical example of the results. Values represent the mean ± S.E.M., n = 5, *P<0.05 vs. control (stimulation with LPS alone). **P<0.05 vs. Mock. Statistical analysis was performed using Student’s *t*-test.

Having demonstrated the CD26-mediated enhancement of mRNA levels, we performed ELISAs to confirm that these enhancements had an effect on the levels of TNF-α and IL-6 protein secreted from TPH-1 cells and human monocytes. As shown in Figure 2, the sCD26-mediated enhancements in mRNA expression are mirrored in the levels of excreted protein. We found that sCD26/DPPIV(+), but not sCD26/DPPIV(−), enhanced the LPS-stimulated secretion of TNF-α protein from TPH-1 cells (Figure 2A). In contrast, both sCD26/DPPIV(+) and sCD26/DPPIV(−) enhanced the LPS-stimulated secretion of IL-6 protein (Figure 2B) from TPH-1 cells. Consistent with these results, addition of the specific DPPIV inhibitor sitagliptin (1 M) inhibited the sCD26/DPPIV(+)-mediated enhancement of LPS-stimulated excretion of TNF-α from THP-1 cells, but had no effect on the LPS-stimulated excretion of IL-6 protein. We also found that sCD26/DPPIV(+), but not sCD26/DPPIV(−), enhanced the LPS-stimulated secretion of TNF-α protein (Figure 2C) from human monocytes. Once again, addition of sitagliptin (1 M) inhibited the sCD26/DPPIV(+)-mediated enhancement of LPS-stimulated TNF-α secretion (Figure 2C). Both sCD26/DPPIV(+) and sCD26/DPPIV(−) enhanced the LPS-stimulated secretion of IL-6 protein from human monocytes, and addition of sitagliptin (1 M) did not have a significant effect (Figure 2D).

### sCD26 Enhances LPS-stimulated Expression of Key Signal Transduction Proteins

**Figure 3:**
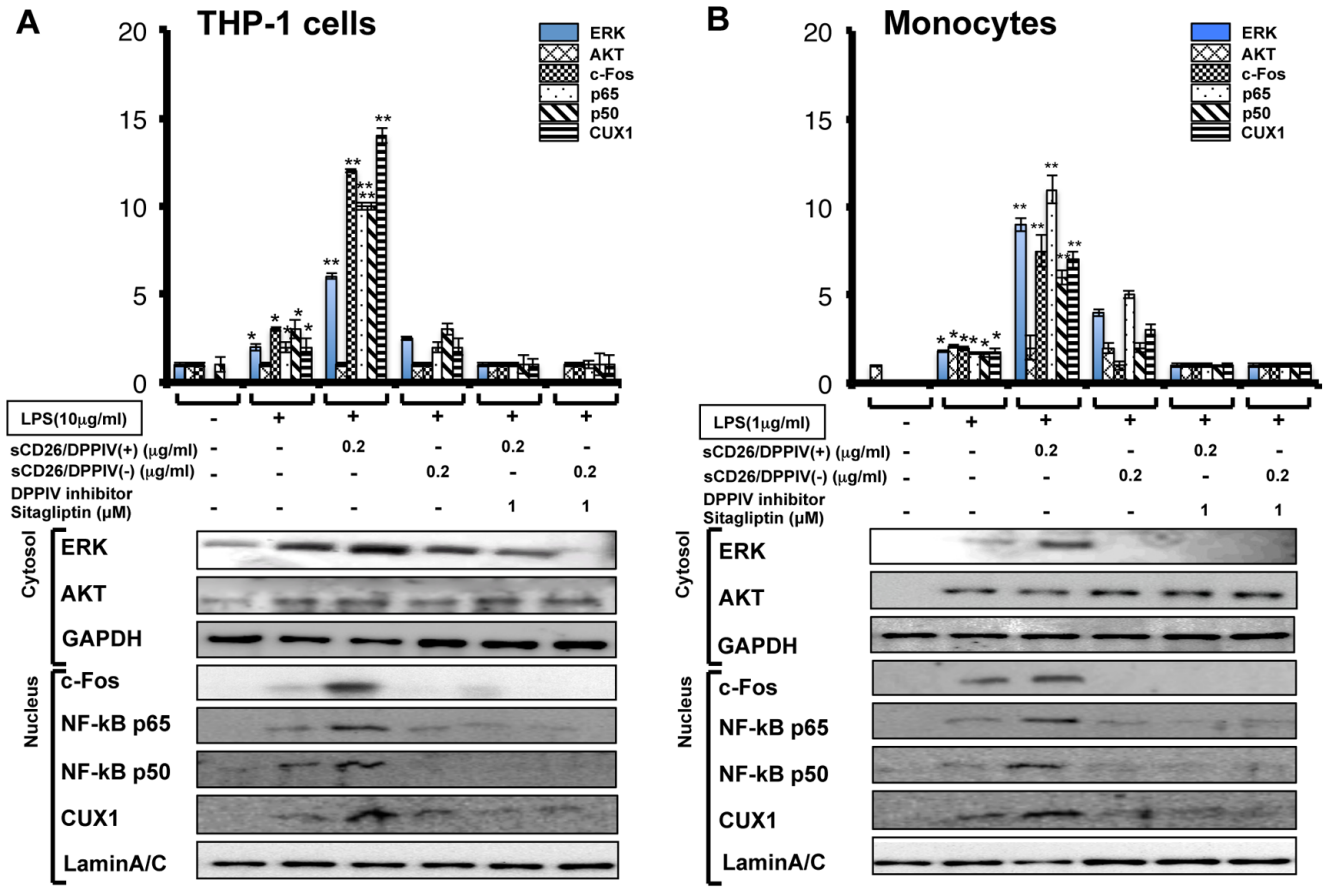
Effects of LPS and sCD26/DPPIV(+) or sCD26/DPPIV(−) stimulation of THP-1 cells and monocytes on ERK1/2, AKT, c-Fos, NF-κB p65, NF-κB p50, and CUX1 protein expression, assessed by Western blotting. A, B. Effects of stimulating THP-1 cells with a combination of LPS and sCD26/DPPIV(+) or sCD26/DPPIV(−) on ERK1/2, AKT, c-Fos, NF-κB p65, NF-κB p50, and CUX1 protein expression with or without the DPPIV inhibitor sitagliptin. THP-1 cells (1×10^5^) were incubated in culture medium with or without LPS, with or without various concentrations of sCD26/DPPIV(+) or sCD26/DPPIV(−), and with or without the DPPIV inhibitor sitagliptin (1 µM). The cells were then cultured for the indicated periods. Cytoplasmic and nuclear fractions were prepared as described in the Methods section and subjected to Western blotting. Each panel shows a typical example of the results. Values represent mean ± S.E.M., n = 3, *P<0.05 vs. control. C, D. Effects of stimulating monocytes with a combination of LPS and sCD26/DPPIV(+) or sCD26/DPPIV(−) on ERK1/2, AKT, c-Fos, NF-κB p65, NF-κB p50, and CUX1 protein expression with or without the DPPIV inhibitor sitagliptin (1 µM). Monocytes (1×10^5^) were incubated in culture medium with or without LPS, with or without various concentrations of sCD26/DPPIV(+) or sCD26/DPPIV(−), and with or without the DPPIV inhibitor sitagliptin (1 µM). The cells were then cultured for the indicated periods. Cytoplasmic and nucleic fractions were prepared as described in the Methods section and subjected to Western blotting. Each panel shows a typical example of the results. Values represent mean ± S.E.M., n = 3, *P<0.05 vs. control. Statistical analysis was performed using Student’s *t*-test.

It is well known that LPS stimulation can increase the levels of ERK, c-Fos, NF-κB, and CUX-1 protein in monocytic cells. We performed Western blotting to detect CD26-mediated enhancement of LPS-stimulated expression of these key signal transduction proteins. We found that sCD26/DPPIV(+) enhanced the LPS-stimulated levels of ERK, c-Fos, NF-κB p65, NF-κB p50, and CUX1 protein in THP-1 cells and human monocytes, but sCD26/DPPIV(−) enhanced the LPS-stimulated levels of these proteins approximately half, or less, compared with sCD26/DPPIV(+) (Figure 3). Consistent with these results, addition of the specific DPPIV inhibitor sitagliptin (1 M) inhibited the enhanced protein expression.

### Effects of sCD26 on the Binding of Signal Transduction Proteins to TNF-α and IL-6 Promoters

**Figure 4:**
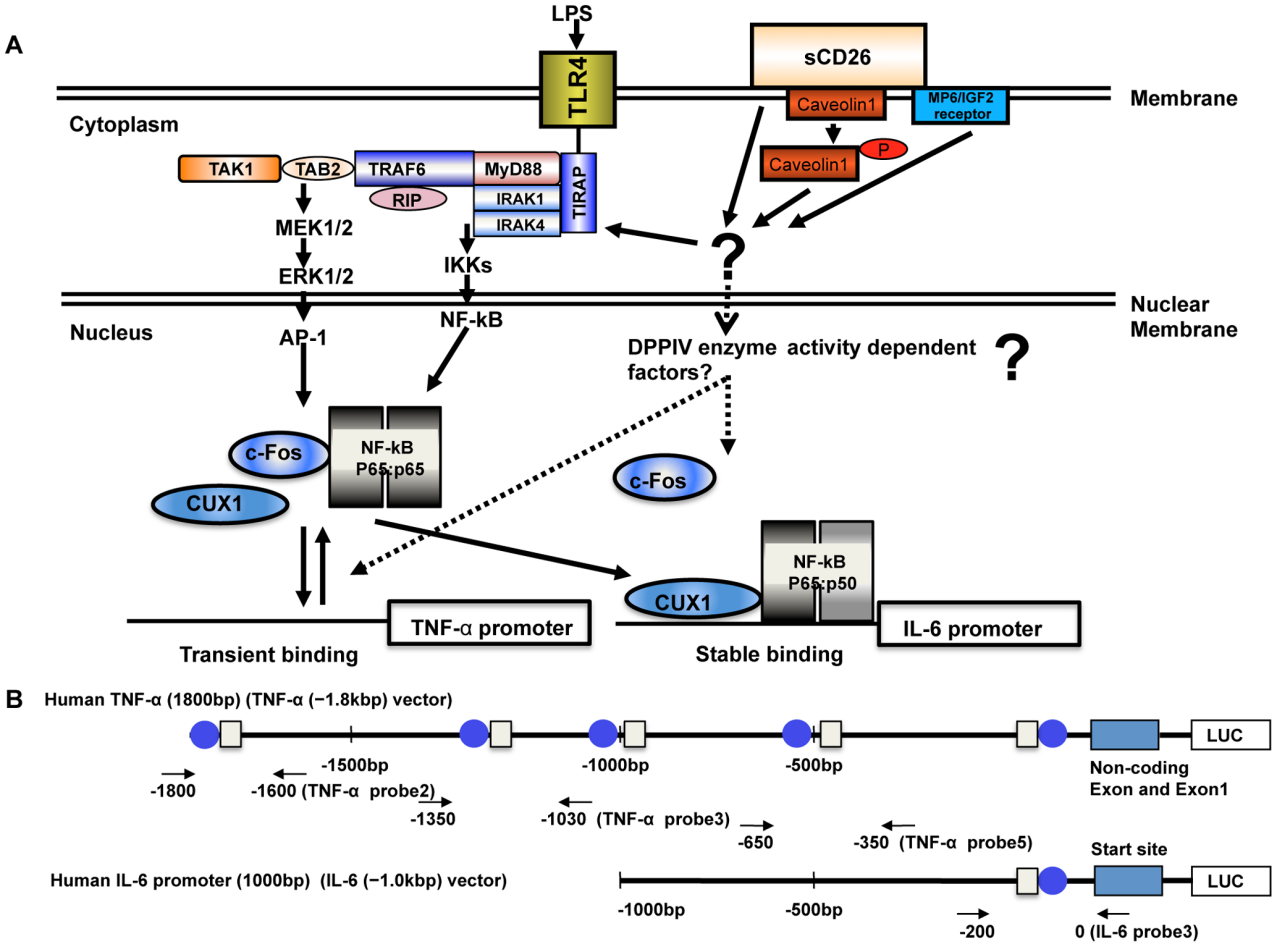
Model for differential regulation of TNF-α and IL-6 gene stimulation by LPS and sCD26/DPPIV(+) or sCD26/DPPIV(−). Stimulation of cells through TLR4 complexes and sCD26 results in ERK1/2, c-Fos, NF-κB, and CUX1 (arrows). c-Fos, NF-κB, and CUX1 transiently bind to the TNF-α gene promoter but stably bind to the IL-6 gene promoter. sCD26 binds caveolin-1 and is internalized by the MP6/IGF receptor 2. The question mark indicates that the sCD26–caveolin interaction or internalization of sCD26 may stimulate IRAK1 in the TLR4 complex. Further investigations are needed. The question mark also indicates that another DPPIV enzyme-dependent factor or factors may exist to modulate the stability of c-Fos, NF-κB, and CUX1 on the TNF-α and IL-6 gene promoters. B. Diagram showing the structures of human TNF-α (−1.8 kb) and IL-6 (−1.0 kb) reporter constructs. The NF-κB sites are indicated by blue circles, and CUX1 sites are indicated by boxes. The positions of ChIP assay probes and primer pairs are indicated by arrows.

**Figure 5:**
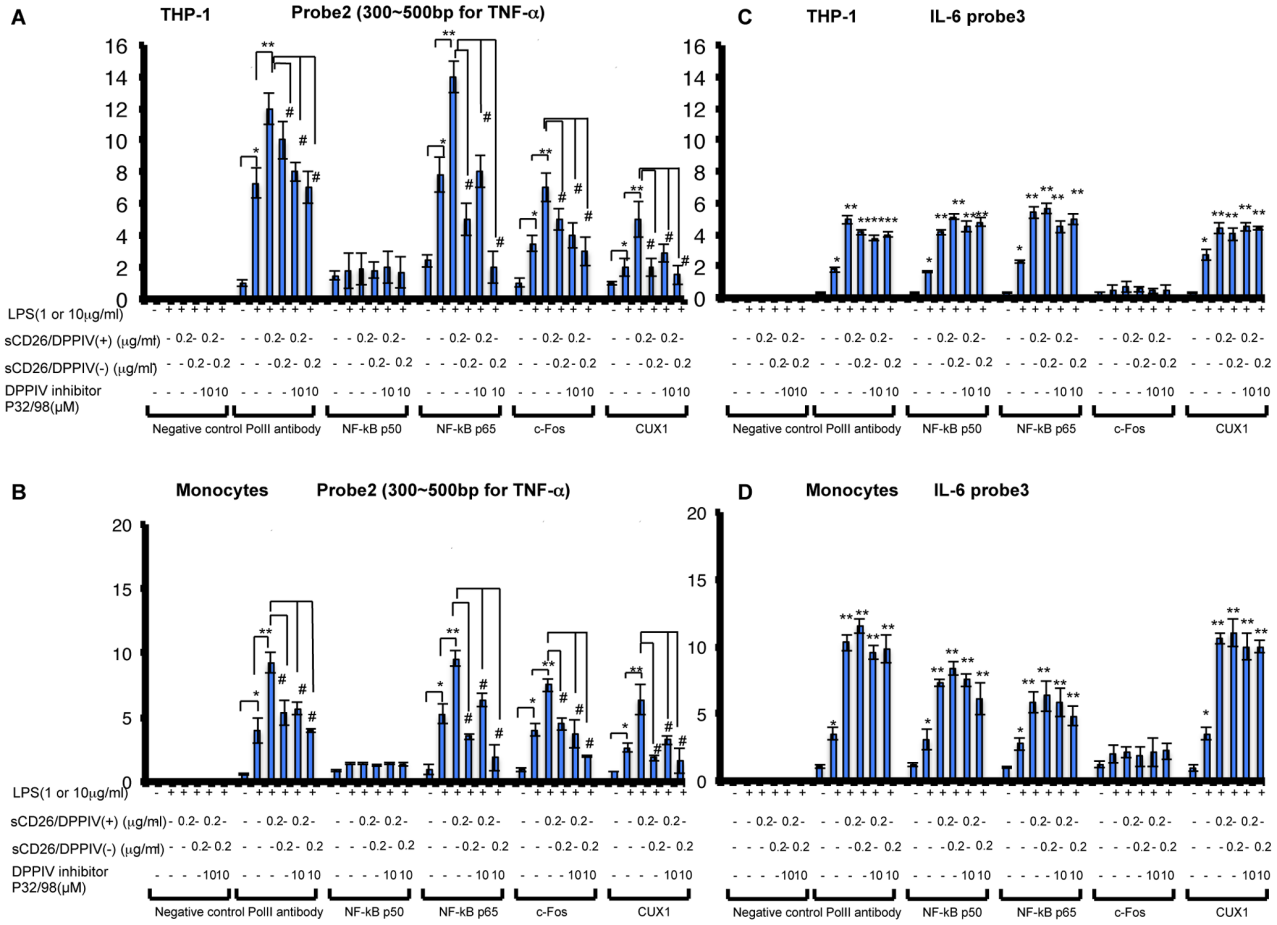
Effects of LPS and sCD26/DPPIV(+) or sCD26/DPPIV(−) stimulation of THP-1 cells and monocytes on the binding affinity of transcription factors with the human TNF-α and IL-6 gene promoters, assessed by ChIP assay. A, C. Effects of stimulating THP-1 cells with a combination of LPS and sCD26/DPPIV(+) or sCD26/DPPIV(−) on the binding affinity of transcription factors to the human TNF-α and IL-6 gene promoters. THP-1 cells (5×10^5^) were incubated in culture medium with or without LPS, with or without various concentrations of sCD26/DPPIV(+) or sCD26/DPPIV(−), and with or without the DPPIV inhibitor P32/98 (10 µM). The cells were then cultured for the indicated periods. They were fixed and subjected to ChIP analysis. Each panel shows a typical example of the results. Values represent mean ± S.E.M., n = 3, *P<0.05 vs. control. **P<0.05 vs. stimulation by LPS alone. ^#^ *P*<0.05 vs. sCD26/DPPIV(+). B, D. Effects of stimulating monocytes with a combination of LPS and sCD26/DPPIV(+) or sCD26/DPPIV(−) on the binding affinity of transcription factors to the human TNF-α and IL-6 gene promoters. Monocytes (5×10^5^) were incubated in culture medium with or without LPS, with or without various concentrations of sCD26/DPPIV(+) or sCD26/DPPIV(−), and with or without the DPPIV inhibitor P32/98 (10 µM). The cells were then cultured for the indicated periods. They were fixed and subjected to ChIP analysis. Each panel shows a typical example of the results. Values represent mean ± S.E.M., n = 3, *P<0.05 vs. control. **P<0.05 vs. stimulation by LPS alone. ^#^ *P*<0.05 vs. sCD26/DPPIV(+). Statistical analysis was performed using Student’s *t-*test.

In the promoter region of TNF-α and IL-6 genes, there are several cis-elements for c-Fos, NF-κB p65, NF-κB p50, and CUX1 (Figure 4B). To investigate the effects of sCD26 on the LPS-stimulated transcriptional regulation of TNF-α and IL-6, we performed ChIP assays. We found that stimulation with a combination of sCD26/DPPIV(+) and LPS increased the binding of c-Fos, NF-κB p65, and CUX1, but not of NF-κB p50, to the TNF-α promoters in THP-1 cells and monocytes, compared with stimulation with LPS alone (Figure 5A, B) Addition of the DPPIV inhibitor sitagliptin (1 M) inhibited the binding of these proteins. While stimulation with a combination of sCD26/DPPIV(−) and LPS did not increase the binding of c-Fos, NF-κB p65, and CUX1 to the TNF-α promoter in THP-1 cells and monocytes, addition of sitagliptin (1 M) decreased binding of these proteins to the TNF-α promoter (Figure 5A, B). The results with the IL-6 promoter were slightly different than those with the TNF-α promoter. In this case, stimulation with a combination of LPS and sCD26/DPPIV(+) or sCD26/DPPIV(−) enhanced the binding of NF-κB p50, NF-κB p65, and CUX1 to the IL-6 promoter compared with stimulation with LPS alone (Figure 5C, D). In this case, addition of the sitagliptin inhibitor (1 M) had no significant effect on the binding.

### Effects of sCD26 on the Activities of the TNF-α and IL-6 Promoters in Monocytic Cells

**Figure 6:**
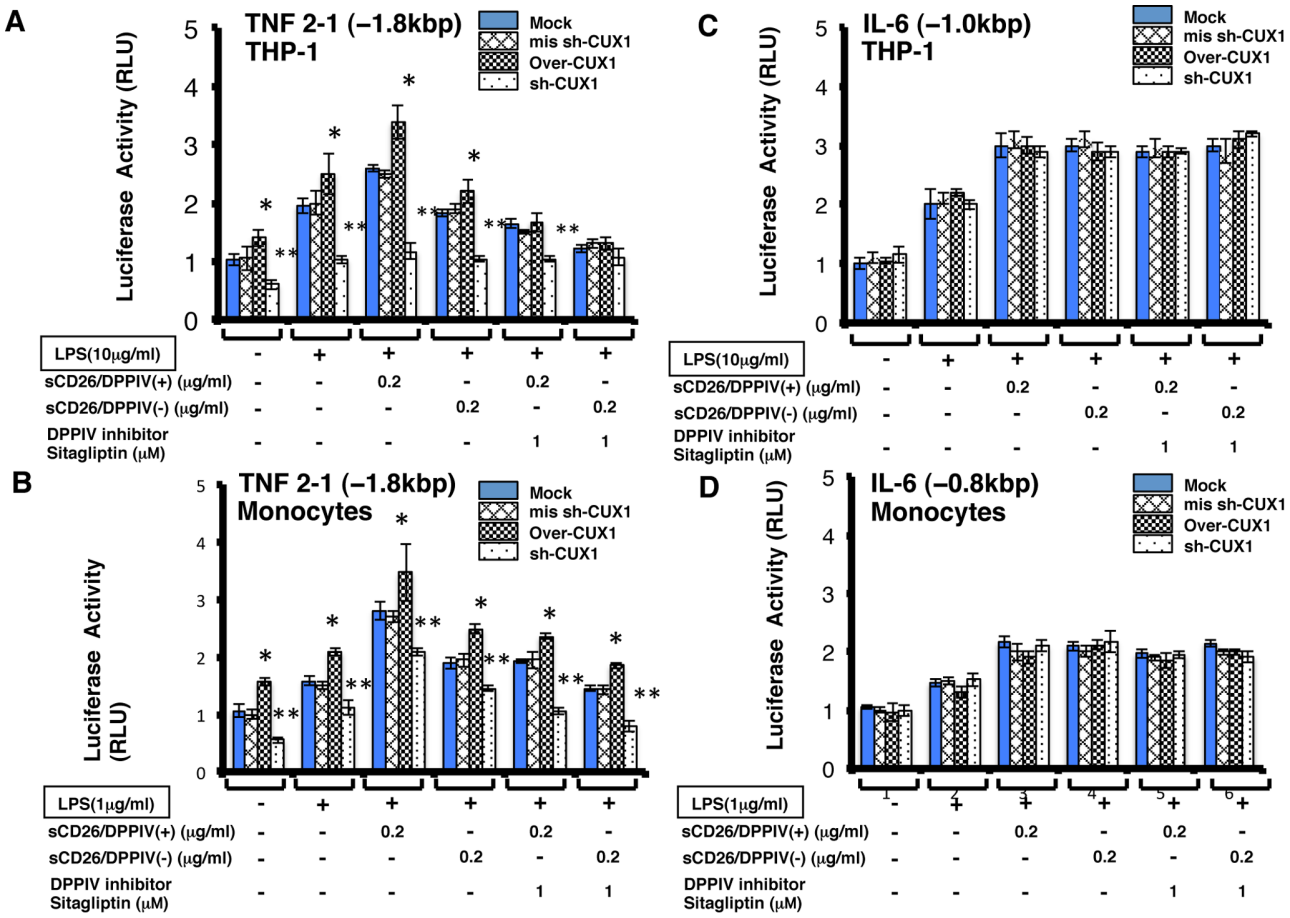
Effects of LPS and sCD26/DPPIV(+) or sCD26/DPPIV(−) stimulation of THP-1 cells and monocytes on the transcriptional activities of the human TNF-α and IL-6 gene promoters. A, C. Effects of stimulating THP-1 cells and monocytes with a combination of LPS and either sCD26/DPPIV(+) or sCD26/DPPIV(−) on the transcriptional activity of the human TNF-α (−1.8 kb) gene promoter. THP-1 cells (5×10^5^) and monocytes (5×10^5^) were incubated in culture medium with or without LPS or various concentrations of either sCD26/DPPIV(+) or sCD26/DPPIV(−), with or without overexpression of CUX1 or sh-CUX1, and with or without the DPPIV inhibitor sitagliptin (1 µM). The cells were cultured for the indicated periods and then subjected to reporter assays. Each panel shows a typical example of the results. Values represent mean ± S.E.M., n = 3, *P<0.05 vs. control. **P<0.05 vs. stimulation with LPS alone. B, D. Effects of stimulating THP-1 cells and monocytes with a combination of LPS and either sCD26/DPPIV(+) or sCD26/DPPIV(−) on the transcriptional activity of the the human IL-6 gene promoter. THP-1 cells and monocytes (5×10^5^) were incubated in culture medium with or without LPS, with or without various concentrations of either sCD26/DPPIV(+) or sCD26/DPPIV(−), with or without CUX1 or sh-CUX1 overexpression, and with or without the DPPIV inhibitor sitagliptin (1 µM). The cells were cultured for the indicated periods and then subjected to reporter assays. Each panel shows a typical example of the results. Values represent mean ± S.E.M., n = 3, *P<0.05 vs. control. **P<0.05 vs. stimulation with LPS alone. Statistical analysis was performed using Student’s *t-*test.

Having demonstrated that sCD26 has an effect on the binding of transcription factors to the TNF-α and IL-6 promoter regions, we performed reporter assays to determine if sCD26 had an effect on the activity of these promoters. We found that stimulation of THP-1 cells or human monocytes with a combination of sCD26/DPPIV(+) and LPS increased the activity of the TNF-α promoter (−1.8 kb) compared with stimulation with LPS alone (Figure 6A, B; Mock). In THP-1 cells, stimulation with a combination of sCD26/DPPIV(−) and LPS did not increase the activity of the TNF-α promoter (−1.8 kb) compared with stimulation with LPS alone (Figure 6A; Mock). Addition of the DPPIV inhibitor sitagliptin (1 M) inhibited the increases in reporter activity mediated by both sCD26/DPPIV(+) and sCD26/DPPIV(−). In contrast, stimulation of TPH-1 cells or monocytes with a combination of LPS and sCD26/DPPIV(+) or sCD26/DPPIV(−) increased the reporter activity of IL-6 (−1.0 kb) compared with stimulation with LPS alone (Figure 6C, D; Mock). Addition of the DPPIV inhibitor sitagliptin (1 M) did not significantly affect the reporter activity mediated by either of the CD26 constructs (Figure 6C, D; Mock).

### CUX1 is a New Transcription Factor that Regulates TNF-α and IL-6 Transcription

Our ChIP results showed that c-Fos (did not bind IL-6), NF-κB p65, NF-κB p50 (did not bind TNF-α), and CUX1 generally bind to the IL-6 promoter more tightly than they do to the TNF-α promoter. In silico analysis of the human TNF-α and IL-6 promoter revealed CUX1 cis-elements near the NF-κB sites (Figure 4B). In addition, Western blotting showed that the level of CUX1 protein increased after monocytic cell stimulation with a combination of sCD26/DPPIV and LPS. Considering these results, we speculated that CUX1 is a new transcription factor that differentially regulates TNF-α and IL-6 transcription in cooperation with c-Fos, NF-κB p65, and NF-κB p50. Therefore, we overexpressed CUX1 and sh-CUX1 in THP-1 cells and monocytes.

In CUX1-overexpressing THP-1 cells and monocytes (Figure 6; over-CUX1), stimulation with a combination of LPS and sCD26/DPPIV(+) or sCD26/DPPIV(−) increased the activity of the TNF-α promoter [TNF 2-1 (−1.8 kb)] compared with the Mock (Figure 6A, B). Addition of the DPPIV inhibitor sitagliptin (1 M) inhibited the activity of TNF-α promoter [TNF 2-1 (−1.8 kb)] under these conditions, similar to that observed with the Mock (Figure 6A, B). In THP-1 cells and monocytes that overexpress sh-CUX1, stimulation with a combination of LPS and sCD26/DPPIV(+) or sCD26/DPPIV(−) decreased the activity of the TNF-α [TNF 2-1 (−1.8 kb)] promoter compared with the Mock (Figure 6A, B; sh-CUX1). Addition of the DPPIV inhibitor sitagliptin (1 M) to these experiments had no effect on the activity of the TNF-α [TNF 2-1 (−1.8 kb)] promoter in THP-1 cells (Figure 6A) but was slightly inhibitory in human monocytes (Figure 6B). Deletion mutants of the TNF-α promoter [TNF-α 3-1 (−1.6 kb), TNF-α 5-1 (−0.8 kb)] showed results similar to those observed with the TNF-α (−1.8 kb) promoter (see Figure S1).

In contrast to the results with the TNF-α promoter, stimulation of THP-1 cells or monocytes that overexpress CUX1 with a combination of LPS and sCD26/DPPIV(+) or sCD26/DPPIV(−) yielded the same IL-6 promoter [IL-6 (−1.0 kb)] activity as the Mock (Figure 6C, D; over-CUX1). Addition of the DPPIV inhibitor sitagliptin (1 M) also had no effect on the activity of the IL-6 (−1.0 kb) reporter in cells overexpressing CUX, similar to that seen with the Mock (Figure 6C, D). These results indicated that CUX1 can bind more tightly to the IL-6 promoter than to the TNF-α promoter.

### Effects of CUX1 on the Secretion of TNF-α and IL-6 from Monocytic Cells

**Figure 7:**
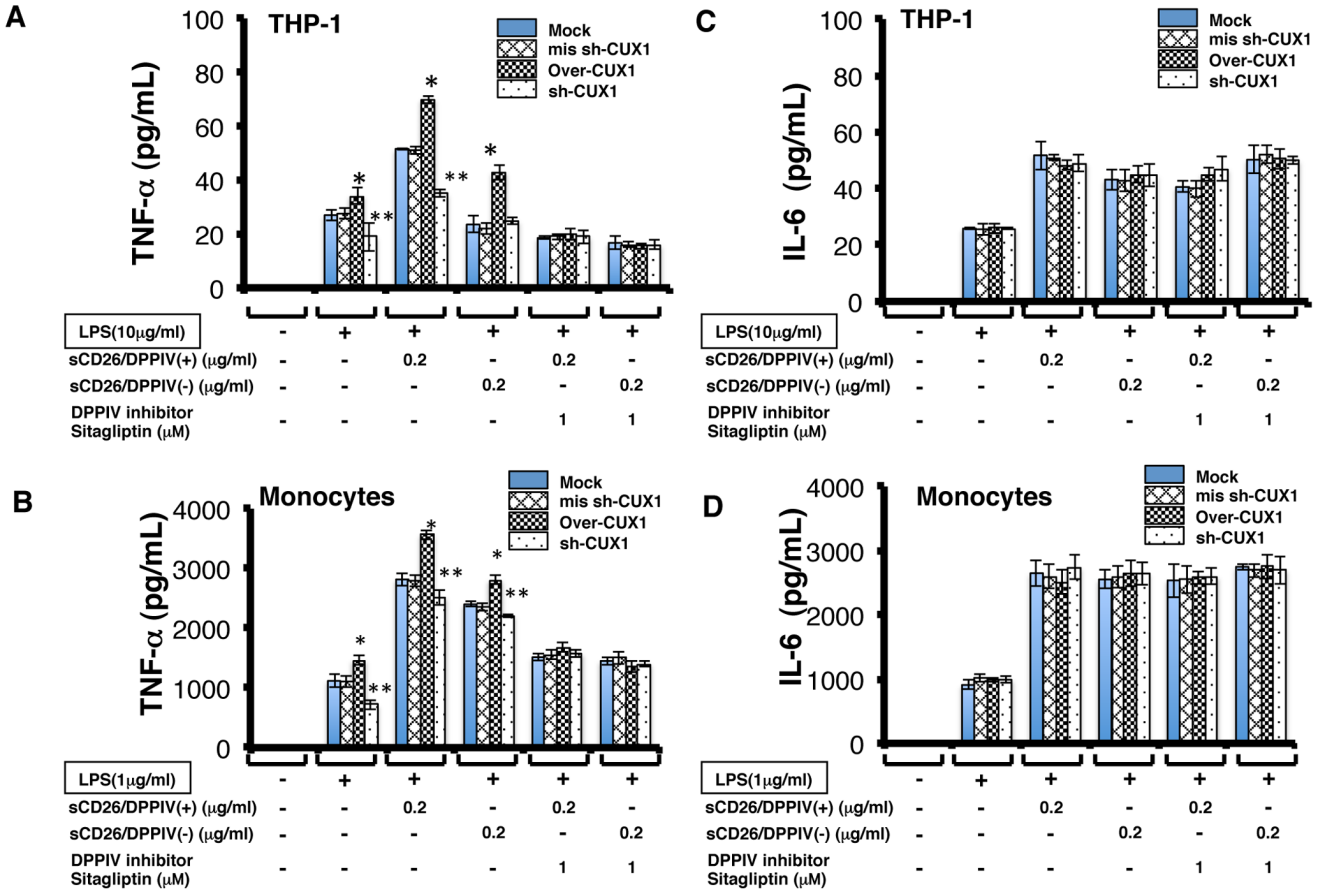
Effects of CUX1 and sh-CUX1 overexpression on TNF-α and IL-6 protein expression in THP-1 cells and monocytes stimulated with LPS and sCD26/DPPIV(+) or sCD26/DPPIV(−), assessed by ELISA. **A, C.** Effects of CUX1 and sh-CUX1 overexpression on TNF-α protein expression in THP-1 cells and monocytes stimulated with a combination of LPS and sCD26/DPPIV(+) or sCD26/DPPIV(−). THP-1 cells (5×10^5^) or monocytes (5×10^5^) were incubated in culture medium with or without LPS, with or without various concentrations of sCD26/DPPIV(+) or sCD26/DPPIV(−), with or without CUX1 or sh-CUX1 overexpression, and with or without the DPPIV inhibitor sitagliptin (1 µM). The cells were cultured for the indicated periods, and serum was then obtained from the well and subjected to ELISA analysis. Each panel shows a typical example of the results. Values represent mean ± S.E.M., n = 5, *P<0.05 vs. control. **P<0.05 vs. stimulation with LPS alone. B, D. Effects of CUX1 and sh-CUX1 overexpression on IL-6 protein expression in THP-1 cells and monocytes stimulated with a combination of LPS and either sCD26/DPPIV(+) or sCD26/DPPIV(−). THP-1 cells (5×10^5^) or monocytes (5×10^5^) were incubated in culture medium with or without LPS, with or without various concentrations of sCD26/DPPIV(+) or sCD26/DPPIV(−), with or without CUX1 or sh-CUX1 overexpression, and with or without the DPPIV inhibitor sitagliptin (1 µM). The cells were cultured for the indicated periods, and serum was then obtained from the well and subjected to ELISA. Each panel shows a typical example of the results. Values represent mean ± S.E.M., n = 5, *P<0.05 vs. control. **P<0.05 vs. stimulation with LPS alone. Statistical analysis was performed using Student’s *t*-test.

To investigate the effects of CUX1 overexpression on the secretion of TNF-α and IL-6 from THP-1 cells and monocytes stimulated by a combination of LPS and CD26, we overexpressed CUX1 and sh-CUX1 in these cells. Stimulation of THP-1 cells or monocytes that overexpress CUX-1 with a combination of LPS and sCD26/DPPIV(+) or sCD26/DPPIV(−) increased the secretion of TNF-α compared with that of cells that do not overexpress CUX1 (Mock) (Figure 7A, B). Addition of the DPPIV inhibitor sitagliptin (1 M) inhibited this increase (Figure 7A, B). Stimulation of THP-1 cells or monocytes that overexpress sh-CUX1 with a combination of LPS and sCD26/DPPIV(+) or sCD26/DPPIV(−) decreased or had no effect on the secretion of TNF-α compared with that of cells that do not overexpress sh-CUX1 (Mock) (Figure 7A, B). Addition of the DPPIV inhibitor sitagliptin (1 M) inhibited these effects (Figure 7A, B).

In contrast, overexpression of CUX1 or sh-CUX1 had no effect on the secretion of IL-6 from THP-1 cells or monocytes stimulated with a combination of LPS and sCD26/DPPIV(+) or sCD26/DPPIV(−) (Figure 7C, D). Addition of the DPPIV inhibitor sitagliptin (1 M) did not change this result (Figure 7C, D).

## Discussion

In this study, we showed that stimulation of cultured THP-1 cells or human monocytes with a combination of sCD26/DPPIV(+) and LPS enhances expression of TNF-α and IL-6 mRNA and production of the corresponding proteins, presumably through TLR4 signaling (Figures 1, 2). Stimulation with a combination of sCD26/DPPIV(+) and LPS upregulates ERK in the cytosol and c-Fos, NF-κB p65, NF-κB p50, and CUX1 in the nucleus (Figures 3, 4). NF-κB p65, c-Fos, and CUX1 transiently bind to the TNF-α promoter and enhance TNF-α transcription in a DPPIV enzyme activity-dependent manner. However, NF-κB p65, NF-κB p50, and CUX1 bind stably and with high affinity to the IL-6 promoter and enhance IL-6 transcription in a DPPIV enzyme activity-independent manner (Figures 4, 5, 6, 7). Recent studies have shown that cAMP differently regulates TNF-α and IL-6 transcription by differential binding affinity of c-Fos, NF-κB p65, and NF-κB p50 to their promoters. From these observations, we assume that the NF-κB p65:p65 homodimer is stable at the NF-κB site (GTGAATTCCC) in the TNF-α promoter. However, c-Fos binding to the NF-κB p65:p65 homodimer results in the release of the homodimer and reduces TNF-α gene activation. In contrast, c-Fos does not bind to the NF-κB p65:p50 heterodimer, resulting in the stable binding of heterodimer to the consensus NF-κB (p50:p65 heterodimer) recognition sequence (GGGRNTTTCC) [14] in the IL-6 gene promoter. Our ChIP assay results showed that NF-κB p50 does not bind to the TNF-α promoter but binds to the IL-6 promoter. NF-κB p65 also binds to the promoters of TNF-α and IL-6 genes. Moreover, c-Fos binds to the TNF-α promoter in a DPPIV enzyme activity-dependent manner but not to the IL-6 promoter (Figure 6). The results of our study are consistent with the results reported by K. Koga et al. [14].

We further demonstrated that CUX1 is a new transcription factor that differently regulates the activities of the TNF-α and IL-6 genes (Figures 6, 7). We found CUX1 sis-elements near the NF-κB sites of the TNF-α and IL-6 gene promoters using in silico analysis (Figure 4B). To verify this new CUX1 function, we overexpressed CUX1 and sh-CUX1 and determined the effects of this overexpression on monocytic cells. Overexpression of CUX1 enhanced TNF-α (−1.8 kb) promoter activity and increased the secretion of TNF-α protein stimulated by LPS in combination with sCD26/DPPIV(+) or sCD26/DPPIV(−) (Figures 6A, B, 7A, B). Overexpression of sh-CUX1 decreased the activity of the TNF-α (−1.8 kb) promoter and the level of secreted TNF-α protein stimulated by the combination of LPS with sCD26/DPPIV(+) or sCD26/DPPIV(−) (Figures 6A, B, 7A, B). However, overexpression of CUX1 or sh-CUX1 has no effect on the activity of the IL-6 (−1.0 kb) promoter or the level of secreted IL-6 protein stimulated by LPS in combination with sCD26/DPPIV(+) or sCD26/DPPIV(−) (Figures 6C, D, 7C, D). Differential regulation of TNF-α and IL-6 transcription on stimulation with a combination of LPS and sCD26/DPPIV(+) or sCD26/DPPIV(−) requires CUX1 with c-Fos, NF-κB p65, and NF-κB p50; it is almost identical to the cAMP reaction (Figures 4, 7). Recent knockout mouse analysis showed that CUX1 plays an important role in the immune system [15]. Our study showed that CUX1 induced by LPS [14] in combination with sCD26 has an important role in the innate immune system through TLR signaling.

Interaction of CD26 with caveolin-1 on antigen-loaded monocytes results in CD86 upregulation, thereby enhancing the subsequent interaction of CD86 and CD28 on T cells to induce antigen-specific T-cell proliferation and activation [7]. In addition, the interaction of CD26 with the mannose-6-phosphate/insulin-like growth factor II receptor plays a role in CD26-induced T-cell costimulation and internalization of CD26 [10]. The mechanism of sCD26 uptake during LPS stimulation is yet to be determined. Accumulating evidence suggests that DPPIV enzyme activity plays an essential role in CD26-mediated T cell costimulation as well as in T cell immune reactions [16]. Previous studies suggested that several potential scenarios may explain the observed effects of DPPIV on immune activation [6], [17], [18], [19], [20], [21]. It is possible that CD26 exerts its effect through the membrane-bound form, particularly in T cells. It is also possible that the CD26 exerts its effect with the soluble form, in view of the fact that CD26 is actually present in human serum [22]. Previous studies with CD26-transfected Jurkat cells, which demonstrated that the DPPIV activity of CD26 augmented the cellular responses of CD26-transfected Jurkat cells to external stimuli, support the former possibility [6], [22]. In contrast, data demonstrating that recombinant sCD26 with DPPIV enzyme activity enhanced proliferative responses of PBMC to stimulation with the soluble recall antigen TT support the latter scenario. Our results showed that the DPPIV inhibitor sitagliptin does not inhibit the increases in IL-6 mRNA and protein expression but inhibits the increases in TNF-α mRNA and protein expression (Figures 1, 2). These results indicate that unknown factors, which are induced in a DPPIV enzyme activity-dependent manner, may exist and affect the binding affinity of c-Fos, NF-κB p65, NF-κB p50, and CUX1 to the promoters. Previous reports demonstrated that CD26 regulates immune responses by cleaving selected chemokines at the N terminus to modify their biological functions [17], [21], [23]. Chemokines processed by the DPPIV enzyme activity exhibited lower chemotactic potency, impaired signaling effects, and altered receptor specificity [17], [21]. In view of its ability to cleave selected biological factors as a serine protease, CD26 may exert its effect by cleavage of certain factors that may, in turn, affect the upregulation of TNF-α and IL-6. Further studies are required for the isolation and characterization of CD26-associated factors responsible for regulating the expression of TNF-α and IL-6 (Figure 4).

In conclusion, we propose that immune cell stimulation by a combination of LPS and sCD26 plays an important regulatory role in innate immune systems. The induction of CUX1 proteins could be therapeutic for preventing acute and chronic inflammatory diseases, such as septic shock or autoimmune diseases.

## Supporting Information

Figure S1 Effects of LPS and sCD26/DPPIV(+) or sCD26/DPPIV(–) stimulation of THP-1 cells and monocytes on the transcriptional activities of the human TNF-α deletion mutants [TNF-α 3-1 (−1.6 kb), TNF-α 5-1 (−0.8 kb)].
(TIF)
