# Supplementary figures and images for: Soluble CD26/Dipeptidyl Peptidase IV Enhances the Transcription of IL-6 and TNF-α in THP-1 Cells and Monocytes
Source: PLoS One. 2013 Jun 21;8(6):e66520. doi: 10.1371/journal.pone.0066520 (PMC3689814; doi:10.1371/journal.pone.0066520)

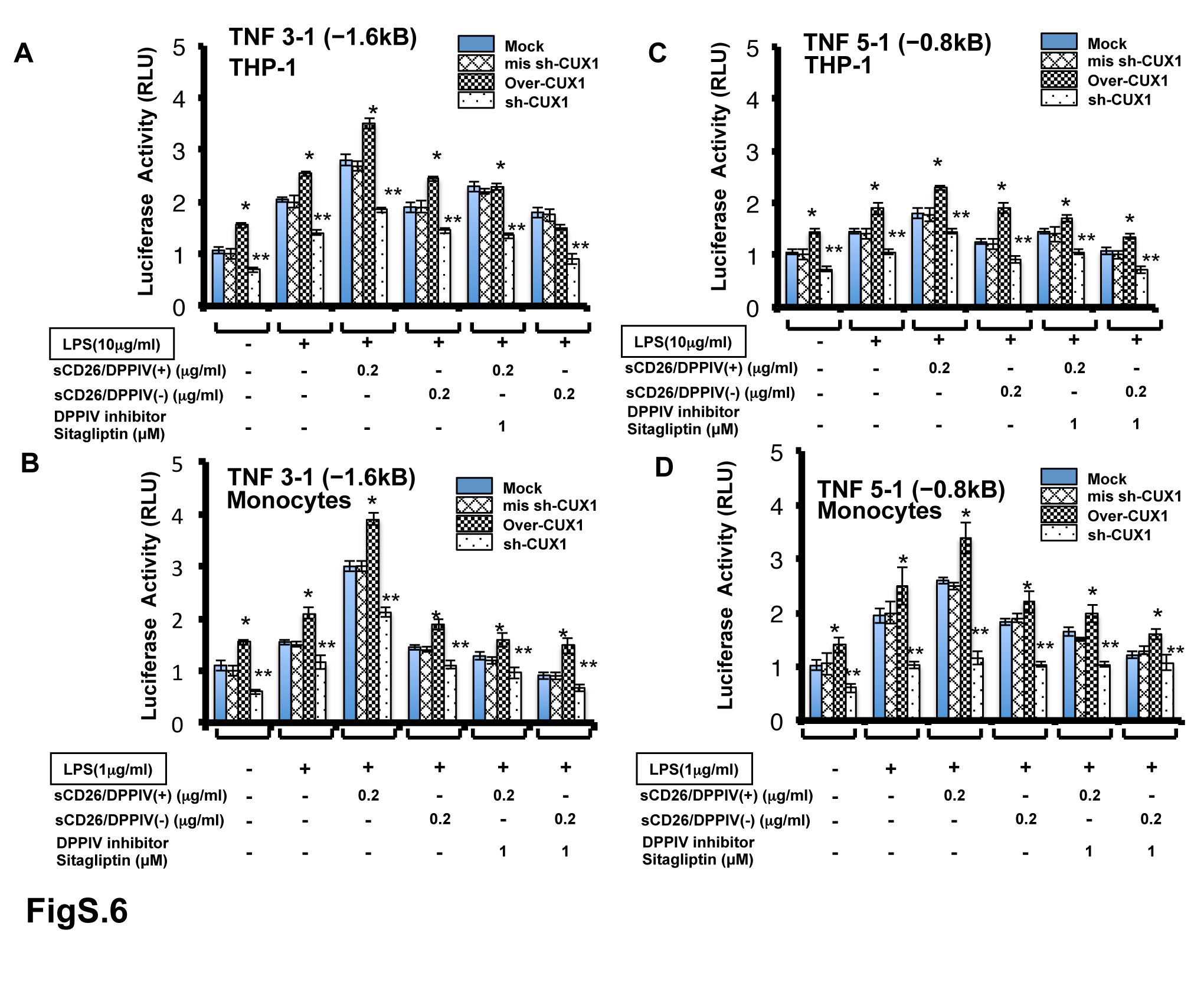

Supplement: Figure S1 — Effects of LPS and sCD26/DPPIV(+) or sCD26/DPPIV(–) stimulation of THP-1 cells and monocytes on the transcriptional activities of the human TNF-α deletion mutants [TNF-α 3-1 (−1.6 kb), TNF-α 5-1 (−0.8 kb)]. (TIF) [file pone.0066520.s001.tif]
